# NIRF Optical/PET Dual-Modal Imaging of Hepatocellular Carcinoma Using Heptamethine Carbocyanine Dye

**DOI:** 10.1155/2018/4979746

**Published:** 2018-03-08

**Authors:** Caiqin Zhang, Yong Zhao, Ningning Zhao, Dengxu Tan, He Zhang, Xue Chen, Hai Zhang, Jiaze An, Changhong Shi, Mengbin Li

**Affiliations:** ^1^Laboratory Animal Center, The Fourth Military Medical University, Xi'an, Shaanxi 710032, China; ^2^Department of Hepatobiliary and Pancreaticosplenic Surgery, Xijing Hospital, The Fourth Military Medical University, Xi'an, Shaanxi 710032, China; ^3^State Key Laboratory of Cancer Biology and Xijing Hospital of Digestive Diseases, The Fourth Military Medical University, Xi'an, Shaanxi 710032, China

## Abstract

Combining near-infrared fluorescence (NIRF) and nuclear imaging techniques provides a novel approach for hepatocellular carcinoma (HCC) diagnosis. Here, we report the synthesis and characteristics of a dual-modality NIRF optical/positron emission tomography (PET) imaging probe using heptamethine carbocyanine dye and verify its feasibility in both nude mice and rabbits with orthotopic xenograft liver cancer. This dye, MHI-148, is an effective cancer-specific NIRF imaging agent and shows preferential uptake and retention in liver cancer. The corresponding NIRF imaging intensity reaches 10^9^/cm^2^ tumor area at 24 h after injection in mice with HCC subcutaneous tumors. The dye can be further conjugated with radionuclide ^68^Ga (^68^Ga-MHI-148) for PET tracing. We applied the dual-modality methodology toward the detection of HCC in both patient-derived orthotopic xenograft (PDX) models and rabbit orthotopic transplantation models. NIRF/PET images showed clear tumor delineation after probe injection (MHI-148 and ^68^Ga-MHI-148). The tumor-to-muscle (T/M) standardized uptake value (SUV) ratios were obtained from PET at 1 h after injection of ^68^Ga-MHI-148, which was helpful for effectively capturing small tumors in mice (0.5 cm × 0.3 cm) and rabbits (1.2 cm × 1.8 cm). This cancer-targeting NIRF/PET dual-modality imaging probe provides a proof of principle for noninvasive detection of deep-tissue tumors in mouse and rabbit and is a promising technique for more accurate and early detection of HCC.

## 1. Introduction

Diverse imaging modalities have been used for liver cancer diagnosis, including positron emission tomography-computed tomography (PET/CT) [1], magnetic resonance imaging (MRI) [2], and fluorescence molecular imaging (FMI) [3]. However, these techniques have different characteristics due to the different imaging principles upon which they are based, and each has its own advantages and disadvantages for various physiological parameters; thus, no single modality can provide comprehensive physiological and pathological information on an organism. Nuclear imaging is an attractive modality for cancer detection [4]. PET for nuclear imaging has distinct advantages in the detection of tumors, possessing high tissue penetration and noninvasive properties capable of monitoring the metabolic and molecular characteristics of cancer cells [5]. However, these probes often exhibit shortcomings, such as a short half-life, low spatial resolution, exposure to radiation, and abundant uptake by tissues with high basal metabolic rates, such as the brain [6].

Near-infrared fluorescent (NIRF) imaging has much higher spatial resolution, and it is an appealing method for the diagnosis of early-stage cancer owing to its multidetection capabilities and high sensitivity [7, 8]. It has been reported that a group of NIRF heptamethine carbocyanine dyes exhibit dual imaging and specific tumor accumulation capabilities [9], including IR-780 [10], IR-783 [11], IR-808 [12], and MHI-148 [13]. Such dyes can be directly absorbed by tumor cells rather than normal cells and accumulate in the mitochondria and lysosomes. The activation of the HIF1*α*/OATPs signaling axis in cancer cells is responsible for the tumor-specific uptake of heptamethine carbocyanine dyes; thus, tumors can be identified without chemical modification [11, 13]. After these dyes bind to biomolecules, high intensity fluorescence is excited by chemical modification [14]. Additionally, this novel class of NIRF dyes exhibited no systemic toxicity when mice were given a 100-fold excess of the imaging dose [15]. Despite these advantages, one disadvantage of NIRF for bioimaging is its very low tissue penetration.

Since heptamethine carbocyanine NIRF dyes accumulate specifically in cancer cells, radioisotope-labeled NIRF dyes are feasible alternative tools for the nuclear imaging of tumors [16]. NIRF imaging is described within the context of nuclear imaging technologies that remain the “gold standard” of molecular imaging. PET and optical imaging are an attractive combination to improve the penetration depth of fluorescence light in biological tissue [17, 18]. NIRF/PET contrast agents not only provide detailed spatial resolution in imaging but also allow for the imaging of molecular targets at low concentrations with high sensitivity and enable improved tumor diagnosis.

Hepatocellular carcinoma (HCC) is the most common malignant cancer worldwide [19]. Although a suite of methods has been developed for early detection with early therapeutic intervention, diagnosing HCC early in clinical management is difficult because signs and symptoms often do not appear until the later stages, leading to a poor survival rate for most HCC cases [20, 21]. Additionally, most agents are eventually deposited in the liver to some extent for metabolism after entering and circulating in the body, where they then release signals, obstructing the precise differentiation of HCC from normal/benign liver tissues; this inhibits a firm diagnosis, particularly at early disease stages [22]. HCC tumor heterogeneity further complicates the results of molecular probes that only target a single antigen or metabolic substrate for tumor visualization. Thus, there is an urgent need for the development of appropriate agents with high tumor-targeting specificity for imaging-based early detection and improved prognosis of HCC.

MHI-148 dye exhibits dual imaging and specific tumor accumulation properties. It accumulates at tumor sites in human liver cancer or gastric cancer subcutaneous xenograft mice, exhibiting good specificity [23, 24]. However, its poor penetration limits its ability to detect tumors more than 1 mm deep, and it is difficult to obtain in vivo whole-body images of rabbit or dog tumor models directly. In this study, we tested the efficacy of MHI-148 in recognizing HCC tumors in both a human HCC orthotopic patient-derived tumor xenograft (PDX) model and cell line-derived tumor xenograft (CDX) model. In order to improve the diagnostic effect of MHI-148 on deep tumors, we synthesized a novel NIRF/PET molecular imaging probe, ^68^Ga-labeled NIRF dye MHI-148 (^68^Ga-MHI-148), and tested it in mice and rabbits with liver orthotopic xenograft tumors. This provided a novel specific vector for PET imaging of hepatic tumors.

## 2. Materials and Methods

### 2.1. Cell Lines and Reagents

**Figure 1:**
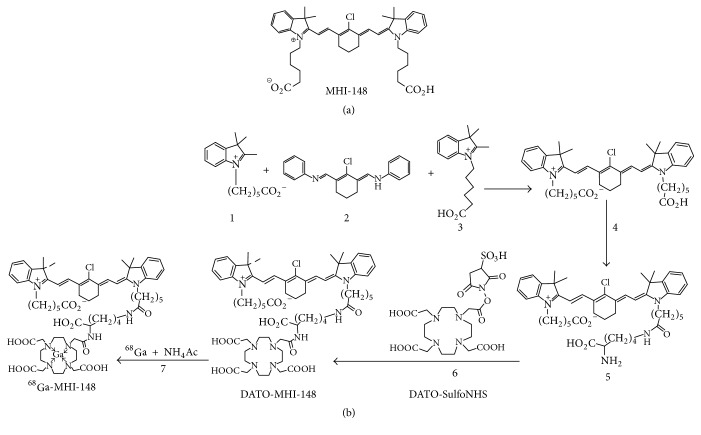
*Design and synthesis of* ^68^ *Ga-MHI-148 probe*. (a) Chemical structure of MHI-148 compound. (b) Synthesis and radiolabeling of NIRF optical/PET dual-modal probe ^68^Ga-MHI-148.

The human HCC Hep3B-3.1 cell line was purchased from the Chinese Academy of Sciences Typical Culture Preservation Committee cell bank and was originally provided by the American Type Culture Collection (ATCC).* OATP3A1*-targeting short hairpin (sh) RNA-carrying lentiviral particles, used for the stable knockdown of this gene, were purchased from Santa Cruz Biotechnology (Santa Cruz, CA, USA). DAPI was purchased from Tiangen (Shanghai, China). Hep3B-3.1 transfected with luciferase cells (Hep3B-3.1-Luc) were derived previously by our lab [22, 24] and cultured in modified Eagle's medium (MEM) supplemented with 10% fetal bovine serum and 1% penicillin/streptomycin (Thermo Scientific, Waltham, MA, USA). Rabbit VX-2 liver tumor tissue was preserved by our lab. The heptamethine carbocyanine dye MHI-148 was kindly provided by Dr. Leland W. K. Chung (Cedars-Sinai Medical Center, Los Angeles, CA, USA) [15], and DOTA-MHI-148 compound was provided by Dr. Dongfeng Pan (The University of Virginia, VA, USA) [25]. The chemical structure of this compound is shown in Figure 1(a).

### 2.2. Synthesis and Radiolabeling of NIRF Dye MHI-148 with ^68^Ga

Radiolabeling was carried out as follows: ^68^Ga/Ga in 400 *μ*l of NH_4_Ac (0.1 N, pH 5.5) was incubated with 50 *μ*g of DOTA-MHI-148 in 200 ml of methanol at 40°C for 30 min. The reaction mixture was purified with high performance liquid chromatography (HPLC) using ^18^C-labeled semipreparative columns. The HPLC parameters were as follows: flow rate, 3 ml/min; gradient, 40% B~100% B in 30 min. The HPLC collection was pooled together, and the acetonitrile was blown by nitrogen flow. The water solution was loaded into a Sep-Pak light ^18^C reverse phase cartridge, and ^68^Ga-MHI-148 was washed down with 1-2 ml of methanol. Finally, the ^68^Ga-MHI-148 residue was dissolved in PBS. The synthesis and radiolabeling of ^68^Ga-MHI-148 probe are shown in Figure 1(b).

### 2.3. Spectroscopic Evaluation

Fluorescence emission spectra were acquired by spectrometer with a variety of excitation wavelengths. The Ga-MHI-148 solution (10 *μ*M, 3 mL) was prepared in 0.1 N PBS buffer (pH 7.45) with 0.25% (v/v) dimethylsulfoxide (DMSO) and was excited with a series of excitation wavelengths to optimize the fluorescence emission spectrum. The optimal emission wavelength of Ga-MHI-148 was 813 nm, the same as MHI-148 (Supplemental 1), which was used for all subsequent studies. This indicated that probe design was not prohibited by conjugation with DOTA-Ga.

^68^Ga-MHI-148 solution (5 *μ*Ci/g mouse) was injected into the tail veins of tumor-bearing mice (*n* = 4). Approximately 50 *μ*L of blood from the contralateral tail vein was collected in capillary tubes at 1 h after probe injection. The radioactivity of both the serum and red blood cells (RBC) was measured and normalized to that of whole blood (100%). The majority of radioactivity was associated with serum, as shown in Supplemental 2. About 90% of the probe bound to the serum instead of RBC.

### 2.4. Animals

Male BALB/c nude mice (6-7 weeks) were purchased from CAVENS (Changzhou, China) and bred in a specific pathogen-free barrier environment at the Laboratory Animal Center of the Fourth Military Medical University (FMMU). Male New Zealand rabbits, weighing 2.3–2.5 kg, were provided by Xi'an Dile Pu Biotechnology (Xi'an, China). Both mice and rabbits were anesthetized with a combination of intravenous ketamine (10 mg/kg) and xylazine (3 mg/kg) and then maintained under isoflurane during surgery and imaging (RC2 rodent gas anesthesia machine). All animal experiments were approved by the animal welfare ethics committee of FMMU (number 16013).

### 2.5. Development of HCC PDX Model

Three liver cancer patient specimens, D68979, D49028, and D67818, were obtained from the Department of Hepatobiliary and Pancreaticosplenic Surgery, Xijing Hospital. Their histological subtypes were moderately differentiated HCC. The use of human tissue specimens in research was approved by the institutional review board (IRB) of FMMU. To establish PDX models, fresh HCC specimens were transplanted subcutaneously into nude mice according to a previously published protocol [23]. Three months later, the implanted tumor was harvested and frozen as 5-mm^3^ fragments in 10% DMSO–Dulbecco's MEM (DMEM) with liquid nitrogen. A part of each tumor was fixed with 4% paraformaldehyde (POM) to be processed as paraffin-embedded tumor blocks and analyzed for morphology against the original patient tumor by hematoxylin and eosin (H&E) staining and immunohistochemical (IHC) analysis. DNA from tumor tissue was extracted for short tandem repeat (STR) analysis. Tumor sections were stained with antibodies specific for HIF1*α* (1 : 100; Abcam, Cambridge, MA, USA), OATP3A1 (1 : 50; GeneTex, Irvine, CA, USA), and AFP (1 : 100; BD Biosciences, San Jose, CA, USA) according to a previously published protocol [22].

### 2.6. Bioluminescence and NIRF Imaging of Tumor Xenograft Models

Whole-body or organ-specific optical imaging was performed at 24 h after the injection of MHI-148 (50 nmol/mouse), using the Caliper Lumina II small animal optical imaging system equipped with NIRF filter sets (excitation/emission, 783/840 nm) [23], and the fluorescence intensity/cm^2^ of tumor (F/T) was calculated. Bioluminescence (BLI) imaging of tumor xenograft was performed after mice received D-luciferin (3 mg/mouse) via intraperitoneal delivery.

### 2.7. NIRF Dye Uptake in Mouse Orthotopic Liver Xenograft Models

Hep3B-3.1-Luc cells (2 × 10^6^) were injected into the livers of nude mice to establish HCC orthotopic xenograft models, as previously reported [24]. In addition, 5-mm^3^ tumor fragments from the D68979, D49028, and D67818 PDX models were implanted into the livers of nude mice [22, 24]. Three weeks later, these mice were injected intraperitoneally with MHI-148 (50 nmol/mouse) and subjected to whole-body NIRF optical imaging. Then, the mice were sacrificed, and NIRF signal intensity was measured in the heart, liver, spleen, lung, and kidney.

### 2.8. PET Imaging of Rabbits and Mice with Liver Orthotopic Transplantation Tumors

Rabbits were anesthetized with a ketamine/xylazine combination, and fresh rabbit VX2 tumor tissue was transplanted into each rabbit's liver, as previously described [24]. Two weeks later, these rabbits and mice with orthotopic liver tumors were intravenously injected with ^68^Ga-MHI-148 at a dose of 0.5 mCi/kg for rabbits and 5 *μ*Ci/g for mice. After 1 h, PET/CT imaging was performed using a Biograph 40 (Siemens, Bonn, GER) for rabbits and Mediso Nano (NSW, Australia) for mice. Nuclear tissue was fixed with 4% formalin for histopathological analysis.

### 2.9. Microarray Data Sets

Four liver cancer DNA microarray data sets—those from Mas et al. [26], Wurmbach et al. [27], Roessler et al. [28], and Ye et al. [29]—were downloaded directly from the Oncomine database by licensed access. These microarray datasets are also publicly available in the Gene Expression Omnibus as GSE14323, GSE6764, GSE14520, and GSE364, respectively [23].

### 2.10. Statistical Analysis

All values are presented as mean ± SEM of at least three independent experiments. Comparisons between Kaplan-Meier curves were performed using the log-rank test. All other comparisons were analyzed by unpaired two-tailed Student's* t*-tests. *p* < 0.05 was considered significant.

## 3. Results

### 3.1. Pathological Analysis of PDX and Clinical Tumor Specimens

**Figure 2:**
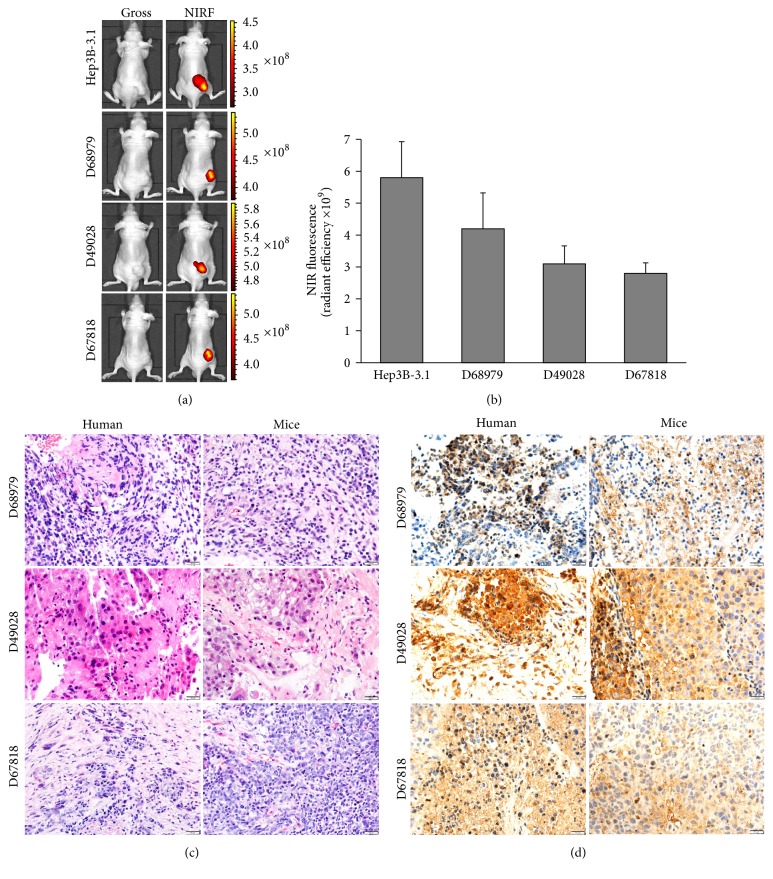
*Uptake of NIRF dye in HCC PDX models*. (a) NIRF imaging of PDX models established by implanting three different fresh human HCC specimens into nude mice subcutaneously. (b) Quantification of NIRF dye uptake in (a) (per cm^2^). Data are presented as the ratio of dye uptake intensity as normalized to that of blank region (mean ± SD, *n* = 5). (c) H&E analyses of tumor tissues derived from both PDX mouse models and original patient samples. (d) AFP expression in tumor tissues derived from both PDX mouse models and original patient samples. Representative images are shown. Original magnification, ×400; scale bars represent 20 *μ*m.

Fresh HCC patient samples were implanted into nude mice subcutaneously to establish PDX models. Three months later, visible tumors formed subcutaneously. Mice bearing xenograft tumors were injected intraperitoneally with MHI-148, and higher intensity NIRF signals were detected at tumor sites compared to those detected with marginal light using whole-body NIRF imaging (Figure 2(a)). The F/T reached 10^9^ (Figure 2(b)). We observed identical histologies in PDX-derived tumor tissues and the original patients' tumor samples using H&E staining, which was accompanied by the strong expression of AFP, a marker highly expressed in HCC (Figures 2(c) and 2(d)). DNA from tumor tissue of PDX models was extracted and subjected to STR analysis using 16 human-specific loci. These loci are derived from alleles selected from across the 23 pairs of human chromosomes and display a high degree of polymorphism. We detected signals at all 16 selected loci (Supplemental 3), and the tumor DNA matched the human-specific loci > 99.99% of the time, confirming that the tumors were human-derived [30]. These results support the idea that tumors from PDX models maintain the morphology and principal molecular characteristics of primary liver tumors.

### 3.2. Uptake of NIRF Dye in Orthotopic HCC Xenograft Models

**Figure 3:**
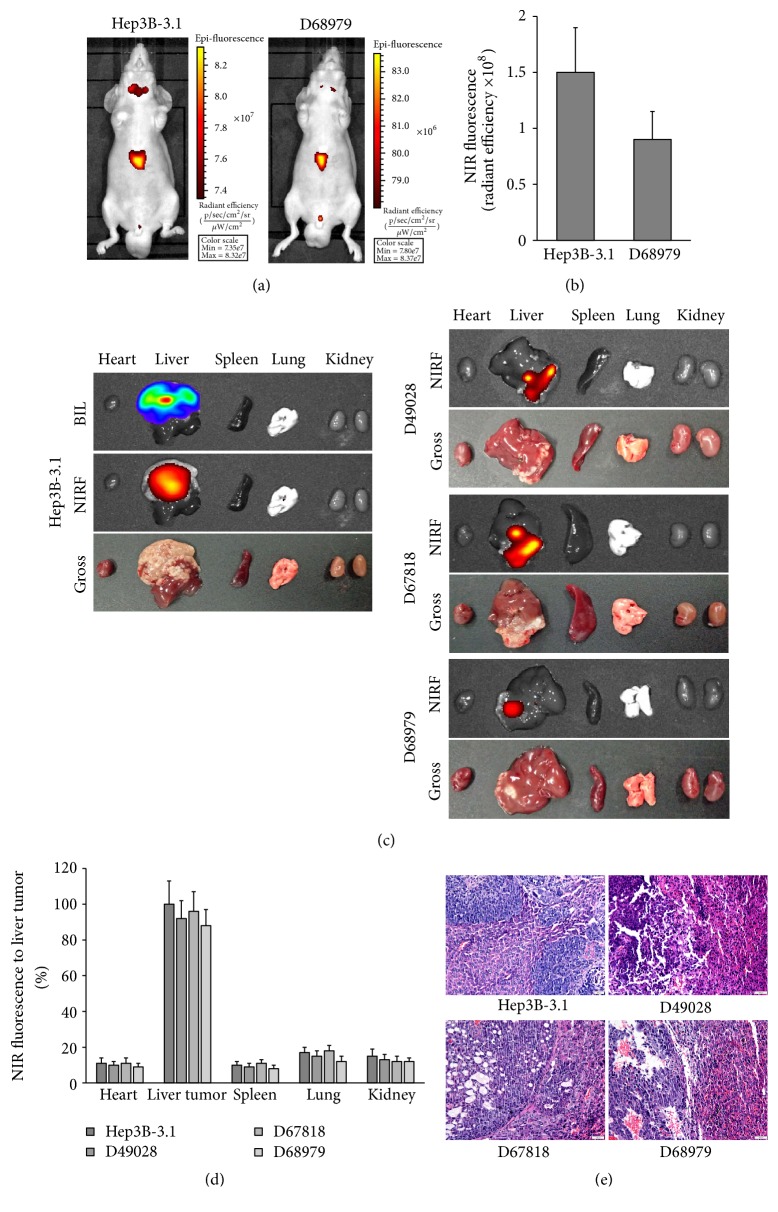
*Preferential uptake and retention of NIRF dye in orthotopic HCC xenograft tumors in mice*. (a) NIRF imaging of Hep3B-3.1 cell-derived tumor xenografts and D68979 PDX tumor xenografts in orthotopic models. HCC Hep3B-3.1-Luc cells and a tumor fragment from a PDX model were implanted into the livers of nude mice. Two weeks later, tumor-emitting signals were captured by NIRF imaging. Representative images are shown. (b) Quantification of NIRF dye uptake in (a) (per cm^2^). Data are presented as the ratio of dye uptake intensity as normalized to that of blank region (mean ± SD, *n* = 5). (c) Ex vivo dual BLI/NIRF imaging of select organs, including the heart, liver, spleen, lung, and kidney, as dissected from mice in (a). (d) Quantification of NIRF signal intensity from the tumor-bearing experimental group in (c). Data are presented as the percentage (mean ± SD, *n* = 5) of signal intensity as normalized to that of liver tumor. Signal intensity in liver tumor is set as 100%. (e) H&E analyses of tumor tissues derived from orthotopic HCC xenograft tumors. Original magnification, ×400; scale bars represent 20 *μ*m.

The subcutaneous implantation of patient tumor tissues into nude mice is a conventional method for the construction of PDX models, and further orthotopic transplantation better simulates the clinical features of liver cancer. In this study, an HCC orthotopic xenograft model was established by injection of Hep3B-3.1 cells and transplantation of PDX model tumor tissues (D68979). After 3 weeks, these mice were intraperitoneally injected with MHI-148 and visualized with whole-body NIRF imaging. MHI-148 exhibited specific accumulation in orthotopic xenografts rather than normal tissue in both PDX-bearing and CDX-bearing mice (Figure 3(a)). NIRF signals were significantly higher in tumor xenografts than in control mice (Figure 3(b)), and the fluorescence intensity reached 10^8^ at tumor sites.

These mice were further sacrificed, and the heart, liver, spleen, lung, and kidney were dissected for NIRF optical imaging. In addition, ex vivo comparisons of biodistributions were performed. The elevated uptake of MHI-148 by tumors in the liver was demonstrated, with a signal intensity more than 5 times higher than that observed in other organs (Figures 3(c) and 3(d)). We also observed obvious white nodules at the signal site, reflecting infiltration and dispersion of growth, as well as limited growth, and tissue morphology and histology were confirmed by H&E staining (Figure 3(e)). That indicated whether the orthotopic transplantation model had been established by HCC cell line or PDX tumor tissue, and tumor formation could be detected by NIRF optical imaging using MHI-148 dye in vivo. In conclusion, we demonstrated that MHI-148 could be used to visualize tumor xenograft in liver cancer PDX models or CDX model with sufficient specificity and sensitivity.

### 3.3. PET Imaging Detection of Orthotopic HCC Tumors in Nude Mice

**Figure 4:**
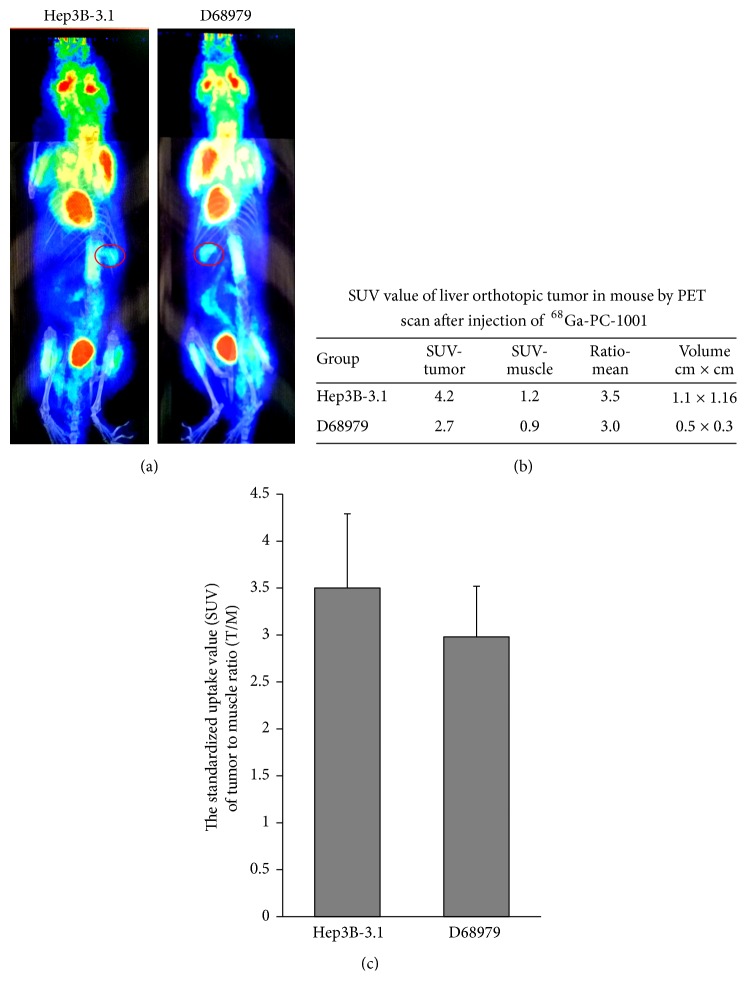
*PET imaging detection of orthotopic HCC tumors in nude mice*. (a) The ^68^Ga-MHI-148/PET conjugate was used to detect HCC orthotopic xenograft models in mice. Strong nuclear signals can be observed at the tumor tissue site (red circle) as compared with that of normal tissue. Representative images are shown. (b) Standardized uptake value (SUV) and tumor volume of live orthotopic tumors in mice according to PET scan after injection with ^68^Ga-MHI-148. (c) Tumor SUV-to-muscle (cancer-adjacent normal muscle tissues) SUV ratios (T/M) are shown.

Mice with HCC orthotopic xenograft tumors detected by NIRF optical imaging were further subjected to PET imaging by tail vein injection of ^68^Ga-MHI-148. Apparent PET signal could be detected at liver tumor sites derived from either HepG3B-3.1 cells or D68979 tissue transplantation (Figure 4(a)). We analyzed the dynamic PET imaging indices, including the standardized uptake value (SUV), as well as the tumor SUV-to-muscle (cancer-adjacent normal tissues) SUV ratios (T/M). The SUV ratios of tumor to normal tissue were 3.5 and 3.0 for HepG3B-3.1-derived and D68979-derived tumors, respectively, and the corresponding tumor sizes were 1.1 cm × 1.16 cm and 0.5 cm × 0.3 cm, respectively (Figures 4(b) and 4(c)). Apparent nuclear signals could be detected at liver sites (Figure 4(a)). The morphology and histology of signal sites were further confirmed with H&E staining (Figure 3(e)).

### 3.4. PET/NIRF Imaging Detection of Orthotopic Liver Tumors in Rabbits

**Figure 5:**
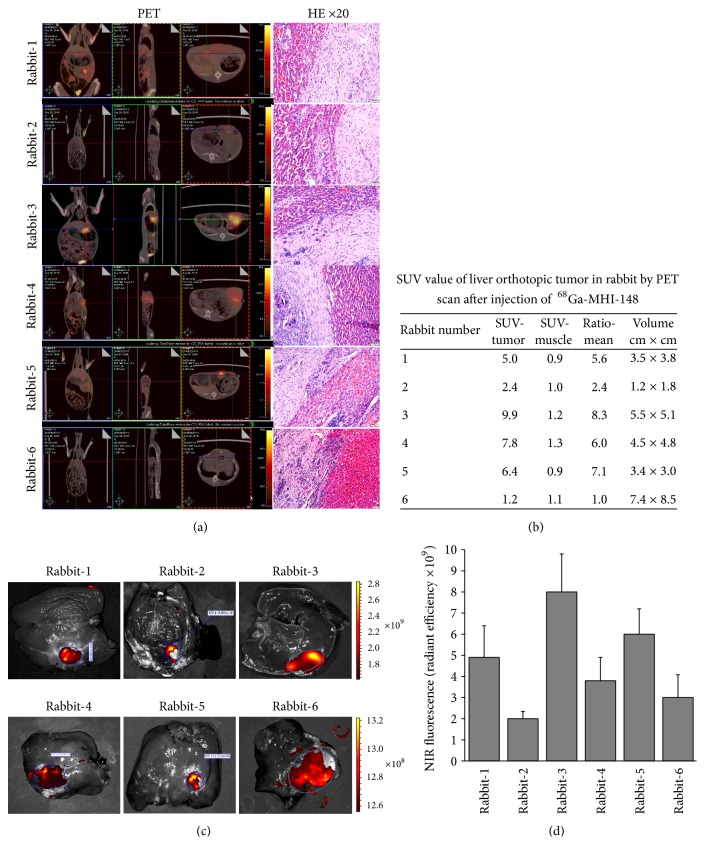
*PET imaging detection of orthotopic liver tumors in rabbits*. (a) PET/CT scan was performed for liver cancer orthotopic xenograft models in rabbit after the injection of ^68^Ga-MHI-148. Strong signals were detected in the liver region (left), and the histology of the targeted cancerous area was confirmed by H&E staining (right). Unlabeled MHI-148 was injected into the sixth rabbit and exhibited a lack of tumor accumulation at tumor sites. Original magnification, ×200; scale bars represent 20 *μ*m. (b) Standardized uptake value (SUV) and tumor volume of live orthotopic tumors in rabbit by PET scan. (c) Ex vivo NIRF imaging of rabbit liver cancer using small animal optical imaging system. (d) Quantification of NIRF intensity within the tumor area (per cm^2^) in (c).

In order to further improve the sensitivity and clinical utility of NIRF dyes for the deep-tissue detection of cancers, we modified the NIRF dye by conjugating it with a positron-emitting radionuclide and tested its ability to detect liver cancer in rabbits. PET scanning was performed 1 h after the injection of ^68^Ga-MHI-148 into the rabbits. The uptake of ^68^Ga-MHI-148 in the liver was clearly observed after a short period (Figure 5(a), left), and the cancerous area with dye accumulation was later confirmed by histology (Figure 5(a), right). Moreover, we analyzed the dynamic PET imaging indices, including the SUV as well as the T/M (cancer-adjacent normal tissues) ratios. The range of T/M ratios for SUV was 2.4–8.3, indicating effective cancer-specific uptake of the radiolabeled NIRF conjugate captured by PET (Figure 5(b)). The corresponding tumor sizes are shown in Figure 5(b), with a minimum size detected of 2.2 cm × 1.8 cm. However, when unlabeled MHI-148 was injected into the rabbit six with liver cancer, no apparent PET signal was detected at tumor sites (Figure 5(a)).

After PET imaging with the ^68^Ga-MHI-148 probe, rabbits bearing VX2 orthotopic liver transplantation tumors were injected with MHI-148 intraperitoneally. After 24 h, livers were surgically removed from anesthetized rabbits and washed with PBS three times, and small animal optical imaging was performed as previously described [24]. A strong NIRF signal was detected at the tumor sites (Figure 5(c)). Quantification of the radiant efficiency showed that the F/T value reached 10^9^ (Figure 5(d)).

### 3.5. Tumor Hypoxia and OATP3A1 Mediate MHI-148 Dye Uptake in HCC

**Figure 6:**
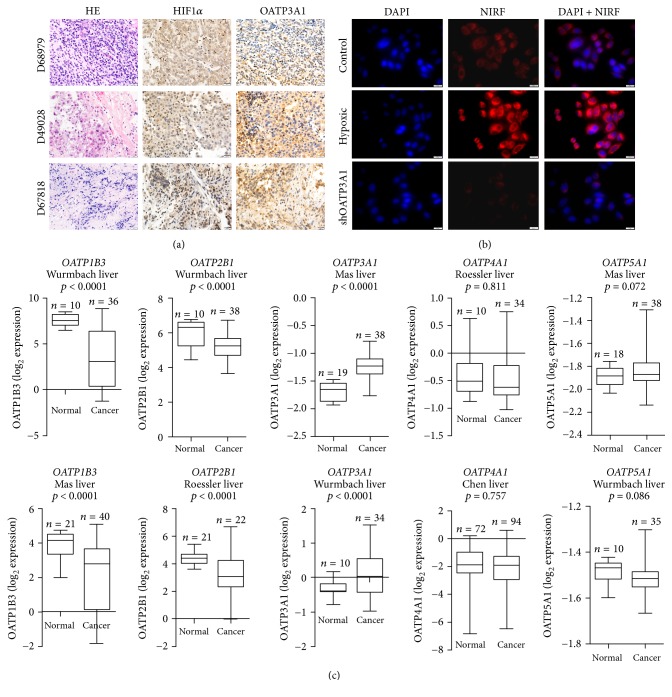
Tumor hypoxia and OATP3A1 mediate MHI-148 dye uptake in HCC. (a) H&E and IHC analyses of HIF1*α* and OATP1B3 protein expression in liver cancer tissues derived from three PDX models. Original magnification, ×400; scale bars represent 20 *μ*m. (b) MHI-148 dye uptake by Hep3B cells with prior exposure to either hypoxia or* OATP3A1*-targeting siRNA. Representative images are shown. Original magnification, 400x; scale bar, 50 *μ*m. (c) Oncomine analysis of select* OATP* transcript levels in different liver cancer data sets (normal versus cancer). ^*∗∗*^*p* < 0.01.

We previously reported that tumor hypoxia and the activation of a group of cell membrane-bound OATPs play important roles in mediating heptamethine carbocyanine dye uptake in different cancer models [13, 14, 31]. To better determine the synergy between hypoxia and OATPs in HCC, we analyzed the expression levels of HIF1*α* and OATP1B3 in three different HCC PDX tissues from an HCC patient sample and found high expression of both proteins (Figure 6(a)). To further determine the effects of hypoxia and OATP3A on MHI-148 uptake, Hep3B cells were treated with MHI-148 (20 *μ*M) for 24 h under 1% O_2_ hypoxic conditions [22]. MHI-148 uptake increased significantly. However, stable knockdown of* OATP3A1* expression in Hep3B cells using* OATP3A1*-targeting siRNA resulted in a 50% decrease in NIRF dye uptake by cancer cells (Figure 6(b)). We further analyzed a number of human liver cancer data sets deposited at the Oncomine website, a cancer microarray database and data-mining platform [26–29]. We found significantly higher expression of* OATP3A1*, low expression of* OATP1B3* and* OATP2B1*, and similar expression of* OATP4A1* and* OATP5A1* in liver cancer patient samples compared to levels in normal tissue samples (Figure 6(c)). These results in sum suggest the mediating roles of both tumor hypoxia and OATP3A1 in NIRF dye uptake by liver cancer cells.

## 4. Discussion

Combining NIRF with PET is a promising technique for overcoming any disadvantages of these modalities, as radionuclide imaging provides better tissue penetration. NIRF/PET multimodal imaging, which integrates the advantages of individual imaging modalities, is popular with researchers [32, 33]. However, few probes are available for liver cancer. Although indocyanine green (ICG), the only FDA-approved NIRF dye for clinical diagnosis, can be used for intraoperative navigation and laparoscopic surgery for liver cancer, it has poor specificity in terms of its binding to tumor cells and can induce strong fluorescence in normal tissues [34, 35]. Moreover, the liver is the main organ for NIRF dye metabolism, which induces a nonspecific background signal with many agents and hinders the development of a probe for liver lesions, limiting the application of conventional NIRF dyes in visualization during tumor surgery. Gao et al. [36] reported a biofunctional probe for the targeting and imaging of liver cancer, but this was only achieved* in vitro*.

The heptamethine carbocyanine dye MHI-148 displays excellent tumor accumulation properties and high quantum yield. It shows low uptake/retention in normal tissue, representing a novel tumor imaging probe [13, 14, 16]. These dyes have been further modified by conjugation with radionuclides and tested for their imaging and accumulation potential in xenograft tumor models. Radioisotope-labeled dyes can not only solve the problem of weak penetration of conventional NIRF dyes, achieving noninvasive imaging, but also be used to determine the extent of tumorigenesis. A NIRF–DOTA conjugate was labeled with ^64^Cu (^64^Cu-PC-1001) for multimodal breast cancer targeted imaging [25]. Sampath et al. [37] synthesized a PET/NIRF agent for the staging of breast cancer and guiding of the subsequent intraoperative resection. In a RANKL-overexpressing LNCaP metastatic prostate tumor xenograft model, NIRF imaging detected two superficial tumors in a mouse after PC-1001 injection, while ^64^Cu-PC-1001 revealed an extra tumor in the same mouse, indicating that the enhanced tissue penetration of gamma radiation enables the detection of deep-tissue tumors [4]. This strategy could allow surgeons to better determine the number, location, and size of liver tumors. The SPECT/NIRF probe ^99^mTc-PC1007 is chemically modified by the NIRF dyes PC-1007 and ^99^mTc, which exhibited preferential accumulation both in human breast cancer MCF-7 cells and in a breast cancer xenograft model [38]. Thus, these NIRFs combined with isotope imaging may allow for novel radionuclide imaging for the early diagnosis of tumors.

We have previously studied the metabolic characteristics of the MHI-148 analogue DZ-1 in nude mice with subcutaneous HCC xenograft tumors [24]. When DZ-1 was injected into nude mice bearing HCC tumors, the fluorescence intensity of the whole mouse body increased rapidly, while the ratio of tumor intensity to background intensity (T/B) increased gradually and reached a peak at 24 h. In this study, PET/CT imaging was performed at 45 min after rabbits or mice with orthotopic liver tumors were injected with ^68^Ga-MHI-148. Thus, apparent PET signal accumulates not only in tumors but also in the primary organs of mice, including the eye, muscles, and lungs (Figures 4 and 5). Over time, however, the background PET signal will decline rapidly, and the tumor SUV-to-muscle SUV (T/M) ratio will increase.

PDX models perfectly maintain the heterogeneity and complexity of original tumors by recapitulating the biological features of gene expression and mutational status at the molecular level, providing an excellent model for cancer research [39]. In this study, MHI-148 compound was successfully used for the optical imaging of liver cancer orthotopic xenograft models, including HCC cell line Hep3B-3.1-derived or HCC PDX-derived transplantation tumors. This dye displayed specific tumor accumulation in all HCC xenograft models tested. After dissection of the mice, ex vivo imaging of mouse organs, including the stomach, liver, spleen, kidney, lung, and heart, was performed to confirm that the fluorescence signal was consistent with the gross morphology of the tumor. The fluorescence intensity of the tumor site was more than 9 times higher than that of the normal tissue. The site of the bioluminescence signal was also consistent with the fluorescence signal in labeled Luc HepG3B-3.1 transplantation tumors.

Compared to the clinical application of the positron-emitting nuclide ^18^F, ^68^Ga labeling is relatively simple and efficient, making it a cost-effective radioisotope [40]. It has a reasonable half-life (67.7 min), and its positron abundance (89%) is suitable for PET imaging [41]. In our previous study, a ^68^Ga-PC-1001 probe was synthesized by conjugating PC-1001 with ^68^Ga for independent PET and fluorescence imaging in canine tumor models. ^68^Ga-PC-1001 achieved outstanding tumor cell labeling capabilities for PET imaging in dogs with spontaneous tumors, with the maximum SUV at the tumor site more than 5 times higher than that in normal tissues and PET/CT images clearly displaying the tumor locations [13]. In this study, we established HCC orthotopic xenograft mice from a tumor cell line (HepG3B-3.1) and PDX tissue (D68979), respectively, and the DOTA-MHI-148 conjugate was incorporated into ^68^Ga (DOTA-MHI-148) for injection into these mice. PET signal was detected at liver tumor sites that were derived from the tumor cell line or tissue transplantation (Figure 4). This indicated that modification of the dye structure with the linker and Ga-DOTA moiety did not affect the tumor-specific property of the dye carrier. We also initially assessed tumor size, with a minimum size detected as small as 0.5 cm × 0.3 cm.

Compared with traditional mouse models, orthotopic liver transplantation tumors in rabbits are generally larger, with deeper tumor sites, and the noninvasive imaging results obtained from rabbits have greater clinical significance. Thus, we further selected five cases of orthotopic transplantation models of rabbit liver cancer for radionuclide imaging. PET images clearly displayed the tumor locations based on the preferential accumulation of ^68^Ga-MHI-148 in tumor tissue versus normal tissue. The tumor site SUV was as high as 8.5, and the smallest tumor size able to be detected was 1.2 cm × 1.8 cm, with the tumor morphology at the signal site confirmed by H&E staining. PET/CT images of sites of NIRF dye-specific aggregation displayed a significant improvement in sensitivity that suggests the preclinical utility of the ^68^Ga-MHI-148 probe for the detection of deep-tissue tumors.

Heptamethine dye primarily accumulates in cancer cells because of their high mitochondrial membrane potential compared with that in normal cells [8]. Both tumor hypoxia and the activation of OATPs are also important mechanisms underlying the uptake of heptamethine dyes by cancer cells [14, 23, 31]. We extended our previous findings to examine whether HCC cells use similar mechanisms to take up and retain NIRF dyes. Altered OATP expression and variants have been implicated in many different types of cancers by several groups [42]. We detected an increase in the mRNA expression of* OATP3A1* in HCC clinical samples and a decrease in the expression of* OATP1B3* and* OATP2B1* compared with levels in normal liver tissue (Figure 6(b)). These patterns are correlated with human liver cancer progression, as examined in a number of human clinical data sets. In our study, we showed that heptamethine carbocyanine dye uptake increased in Hep3B cells under hypoxic conditions but declined in response to* OATP3A1*-targeting siRNA treatment (Figure 6(b)), providing a rationale for the role of tumor hypoxia and OATPs in tracer uptake. Moreover, both HIF1*α* and OATP3A1 were strongly expressed in PDX tumor samples and their original clinical liver tumor specimens (Figure 6(a)). Together, these insights indicate that the activation of OATP3A1 and hypoxic signaling promote NIRF dye uptake in HCC cells.

## 5. Conclusion

In this study, the cancer-targeting NIRF/PET dual-modality imaging probe ^68^Ga-MHI-148 was successfully synthesized and characterized. This probe exhibited cancer-specific targeting and accumulation properties, in both mice with liver orthotopic xenograft tumors (PDX or CDX models) and rabbits with liver orthotopic transplantation tumors. NIRF/PET targeted dual-modality images showed clear tumor delineation after injection. This imaging probe therefore represents a promising method for improving the diagnosis of HCC.

## Supplementary Materials

Supplementary Materials Supplemental Figure  1: spectroscopic analysis. Supplemental Figure  2: blood distribution of 68Ga-MHI-148 at 1 h. Supplemental Figure  3: STR analysis results of tumor DNA.
