# Supplementary material for: NIRF Optical/PET Dual-Modal Imaging of Hepatocellular Carcinoma Using Heptamethine Carbocyanine Dye
Source: Contrast Media Mol Imaging. 2018 Mar 8;2018:4979746. doi: 10.1155/2018/4979746 (PMC5863326; doi:10.1155/2018/4979746)
Supplement: Supplementary Materials — Supplemental Figure 1: spectroscopic analysis. Supplemental Figure 2: blood distribution of 68Ga-MHI-148 at 1 h. Supplemental Figure 3: STR analysis results of tumor DNA. [file 4979746.f1.pdf]

Supplemental Figure 1. Spectroscopic analysis.

A **B**

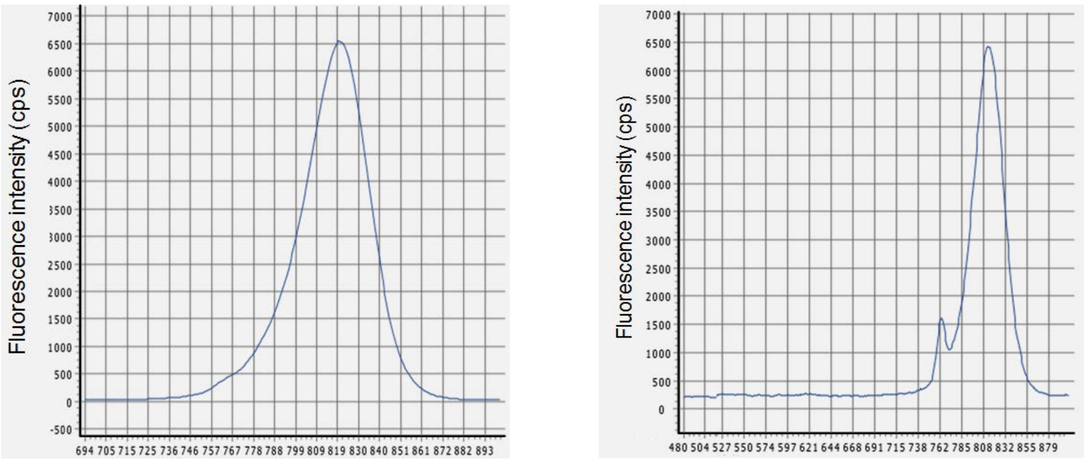

A. Optimization of emission wavelength of MHI-148 is 820 nm. B. Optimization of emission wavelength of Ga-MHI-148 is 813 nm. Optimal excitation wavelength of MHI-148 and Ga-MHI-148 are both 767 nm.

Supplemental Figure 2. Blood distribution of ^{68}Ga-MHI-148 at 1 h.

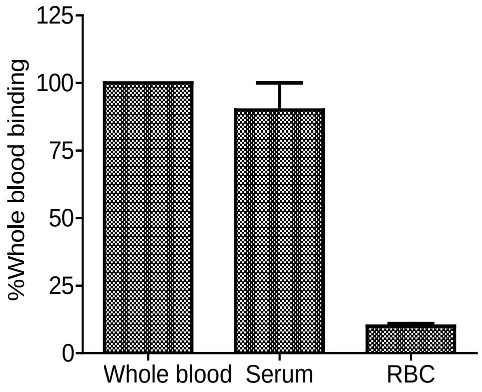

About 90% of the probe bound to the serum instead of red blood cells (n=5).

Supplemental Figure 3. STR analysis results of tumor DNA

Tumor tissue DNA from PDX model (D68979, D49028 and D67818) was subjected to STR analysis. All 16 human-specific loci were detected signals, and the tumor DNA matched the human-specific loci >99.99% of the time, confirming that the tumors were human-derived.

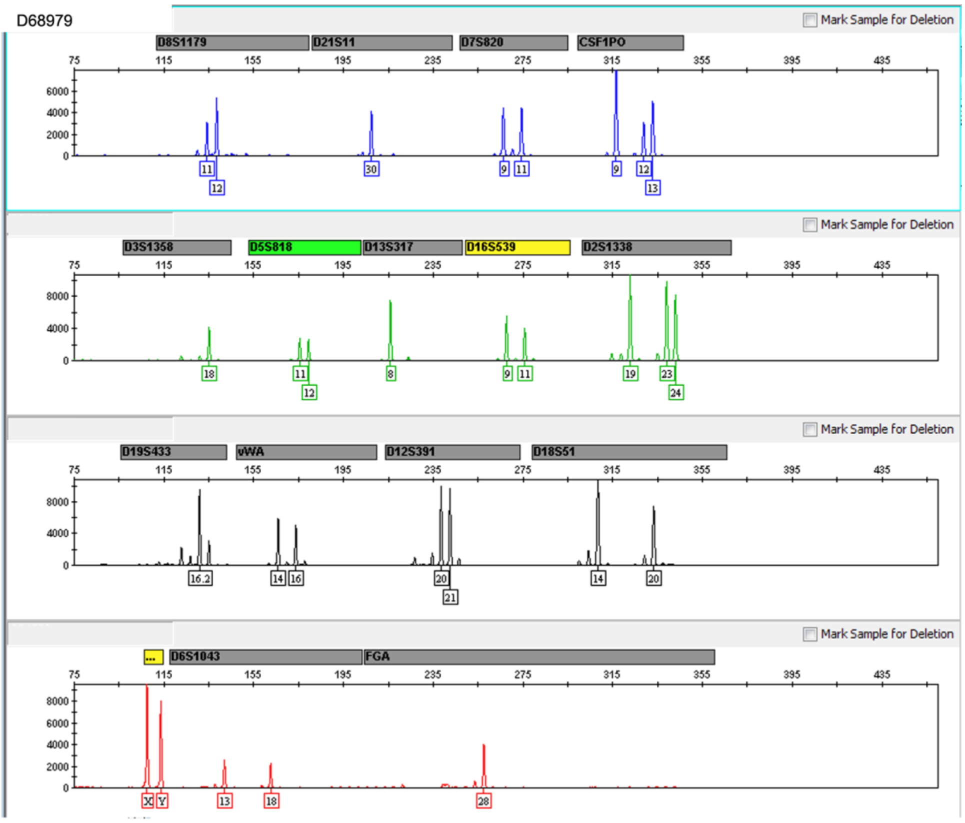

D68979

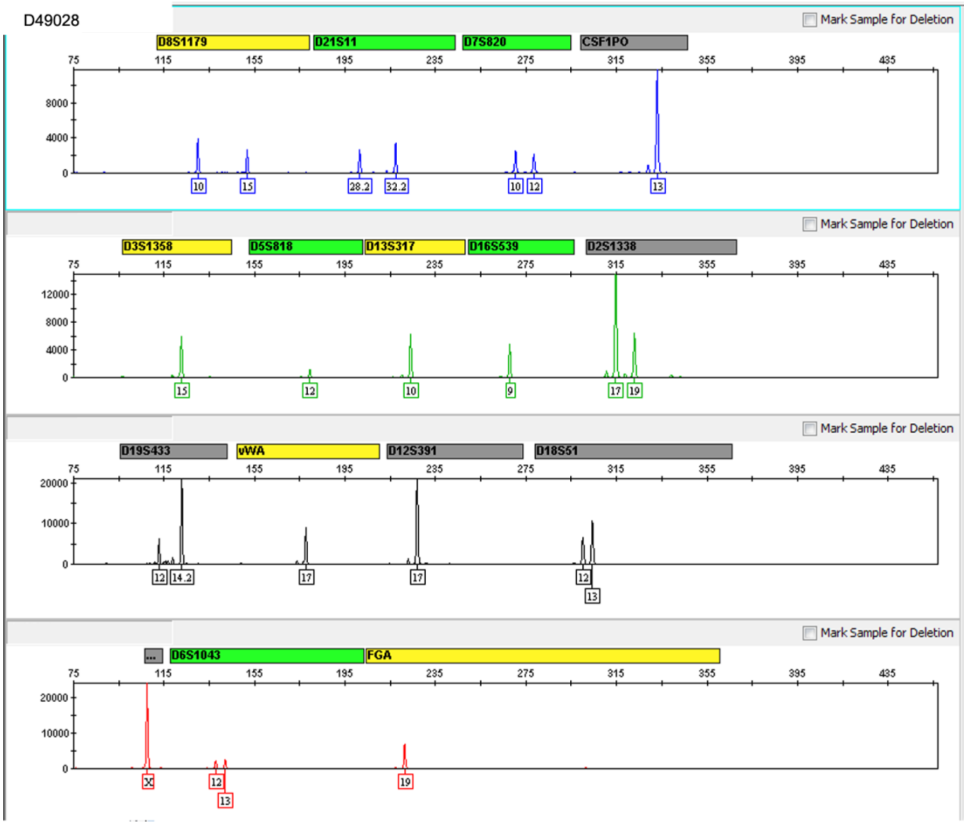
D49028
Mark Sample for Deletion
D8S1179
D21S11
D7S820
CSF1PO
10
15
28.2
32.2
10
12
13
D3S1358
D5S818
D13S317
D16S539
D2S1338
15
12
10
9
17
19
D19S433
vWA
D12S391
D18S51
12
14.2
17
17
12
13
D6S1043
FGA
X
12
13
19

D49028

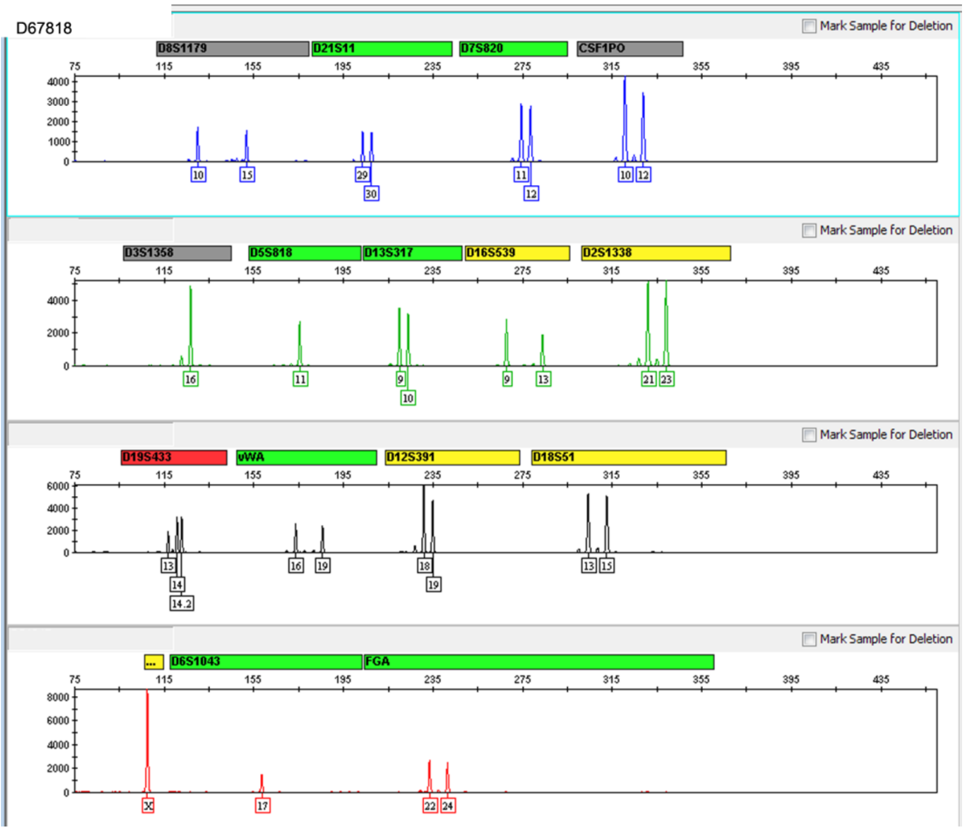
D67818
Mark Sample for Deletion
D8S1179
D21S11
D7S820
CSF1PO
10
15
29
30
11
12
10
12
Mark Sample for Deletion
D3S1358
D5S818
D13S317
D16S539
D2S1338
16
11
9
10
9
13
21
23
Mark Sample for Deletion
D19S433
vWA
D12S391
D18S51
13
14
14.2
16
19
18
19
13
15
Mark Sample for Deletion
D6S1043
FGA
X
17
22
24

D67818
